# Defective Transcriptional Programming of Effector CD8 T Cells in Aged Mice Is Cell-Extrinsic and Can Be Corrected by Administration of IL-12 and IL-18

**DOI:** 10.3389/fimmu.2019.02206

**Published:** 2019-09-18

**Authors:** Mladen Jergović, Heather L. Thompson, Kristin R. Renkema, Megan J. Smithey, Janko Nikolich-Žugich

**Affiliations:** ^1^Department of Immunobiology and the University of Arizona Center on Aging, University of Arizona College of Medicine-Tucson, Tucson, AZ, United States; ^2^Biomedical Sciences Department, Grand Valley State University, Allendale, MI, United States

**Keywords:** aging, cytotoxic T cells, inflammation, cytokines, bacterial infection

## Abstract

In response to infection with intracellular microorganisms, old mice mobilize decreased numbers of antigen-specific CD8 T cells with reduced expression of effector molecules and impaired cytolytic activity. Molecular mechanisms behind these defects and the cell-intrinsic (affecting naïve CD8 T cells themselves) vs. extrinsic, microenvironmental origin of such defects remain unclear. Using reciprocal transfer experiments of highly purified naïve T cells from adult and old transgenic OT-1 mice, we decisively show that the dominant effect is cell-extrinsic. Naïve adult OT-1 T cells failed to expand and terminally differentiate in the old organism infected with *Listeria*-OVA. This defect was preceded by blunted expression of the master transcription factor T-bet and impaired glycolytic switch when T cells are primed in the old organism. However, both old and adult naïve CD8 T cells proliferated and produced effector molecules to a similar extent when stimulated *in vitro* with polyclonal stimuli, as well as when transferred into adult recipients. Multiple inflammatory cytokines with direct effects on T cell effector differentiation were decreased in spleens of old animals, particularly IL-12 and IL-18. Of note, *in vivo* treatment of mice with IL-12 and IL-18 on days 4–6 of *Listeria* infection reconstituted cytotoxic T cell response of aged mice to the level of adult. Therefore, critical cytokine signals which are underproduced in the old priming environment can restore proper transcriptional programming of old naïve CD8 T cells and improve immune defense against intracellular microorganisms.

## Introduction

Infectious diseases remain amongst the leading causes of death in older adults who also often exhibit suboptimal responses to vaccination (1). The immune system exhibits pronounced changes with aging, affecting both innate and adaptive immunity (2), especially the T cell compartment (3) and such changes critically contribute to increased susceptibility to infection with age. However, the precise mechanisms behind most of these changes have not been fully elucidated—particularly not in CD8 cells, which are responsible for clearing intracellular microbial pathogens (4).

We and others have studied CD8 T cell immunity in rodents using microbial pathogens that cause substantial morbidity and mortality in older adults including WNV (5), Chikungunya virus (6), and *Listeria monocytogenes* (7), and have found significant CD8 T cell defects [reviewed in Jergović et al. (8)]. Specifically, old mice infected with the West Nile Virus (WNV) (9), influenza (10), or *Listeria monocytogenes* (Lm) (11) exhibited decreased numbers of Ag-specific effector CD8 T cells, that further exhibited decreased expression of effector molecules, including granzyme B (GrzB), TNF-α, and IFN-γ on a per-cell basis; decreased polyfunctionality (ability to produce multiple effector molecules); and decreased cytolytic activity. While cell transfers of total old and adult CD8 T cells into T and B-cell deficient RAG-KO recipients suggested that old cells mount inferior responses (9), these experiments did not control for the absolute number of naïve T cells in each population and the reciprocal transfers into old recipients were not performed. Therefore, at the present we have no conclusive data on whether defects in an aging host are dominantly CD8 T cell-intrinsic or extrinsic in nature and the mechanisms underlying these deficiencies. Intrinsic T cell defects in CD4 T cells with aging have been reported [reviewed in (12)]. However, the magnitude and quality of the effector T cell response is known to be at least in part determined by T cell extrinsic factors like effective antigen uptake and presentation by antigen presenting cells (APCs), costimulatory, and co-inhibitory signals delivered by APCs and signal 3 cytokines (13).

Cellular metabolism undergoes early and profound changes in the course of naïve T cell activation, shifting from oxidative phosphorylation to glycolysis. That shift and the upregulation of anabolic pathways in T cells have been shown to control the downstream differentiation and effector cascades (14). Yet, we lack information on how this control may change with aging and how the activation-induced signaling cascades and transcriptional pathways interact with cell metabolic processes in old T cells. This is particularly important because these metabolic pathways are critically involved not only in naïve T cell activation, but also in regulation of longevity, health span (15) and immunological memory (16).

To bridge this gap, we probed mechanistic links between transcription, metabolism, and inflammation in the naïve-to-effector CD8 T cell transition with aging. We report here decreases in multiple inflammatory cytokines (IL-12, IL-18, IL-2, IFN-γ) in spleen homogenates, but not serum, of old mice infected with Lm. This decreased inflammatory response was followed by decreased activation of the Th1-specifying master transcriptional regulator, T-bet (T-box expressed in T cells), reduced glucose metabolism, and terminal differentiation of antigen (Ag) specific CD8 T cells primed in the old environment. Of interest, the defects segregated with the old environment—they were no longer observable when old naïve CD8 T cells were primed in an adult organism but were imprinted on adult naïve CD8 T cells primed in an old host. Indeed, differentiation defects were corrected by exogenous IL-12 and IL-18, which were able to selectively and specifically upregulate T-bet, glucose uptake, terminal differentiation, and expansion of Ag specific CD8 T cells both *in vitro* and *in vivo*. Previously, we found that impaired expansion of adult OT-I CD8 in aged recipient mice correlated with functional CD8a+ dendritic cells defects and could be improved, but not fully restored, by exogenous Flt3 ligand treatment (17). Here, we expand on these findings and show that both the expansion and differentiation defects of CD8 T cells in aged mice could be reconstituted to the levels observed in adult mice, using specific cytokine treatments.

## Results

### Impaired Differentiation and Expansion of CD8 T Cells Responding to *Listeria monocytogenes* in Old Mice Is Driven by the Priming Environment

**Figure 1:**
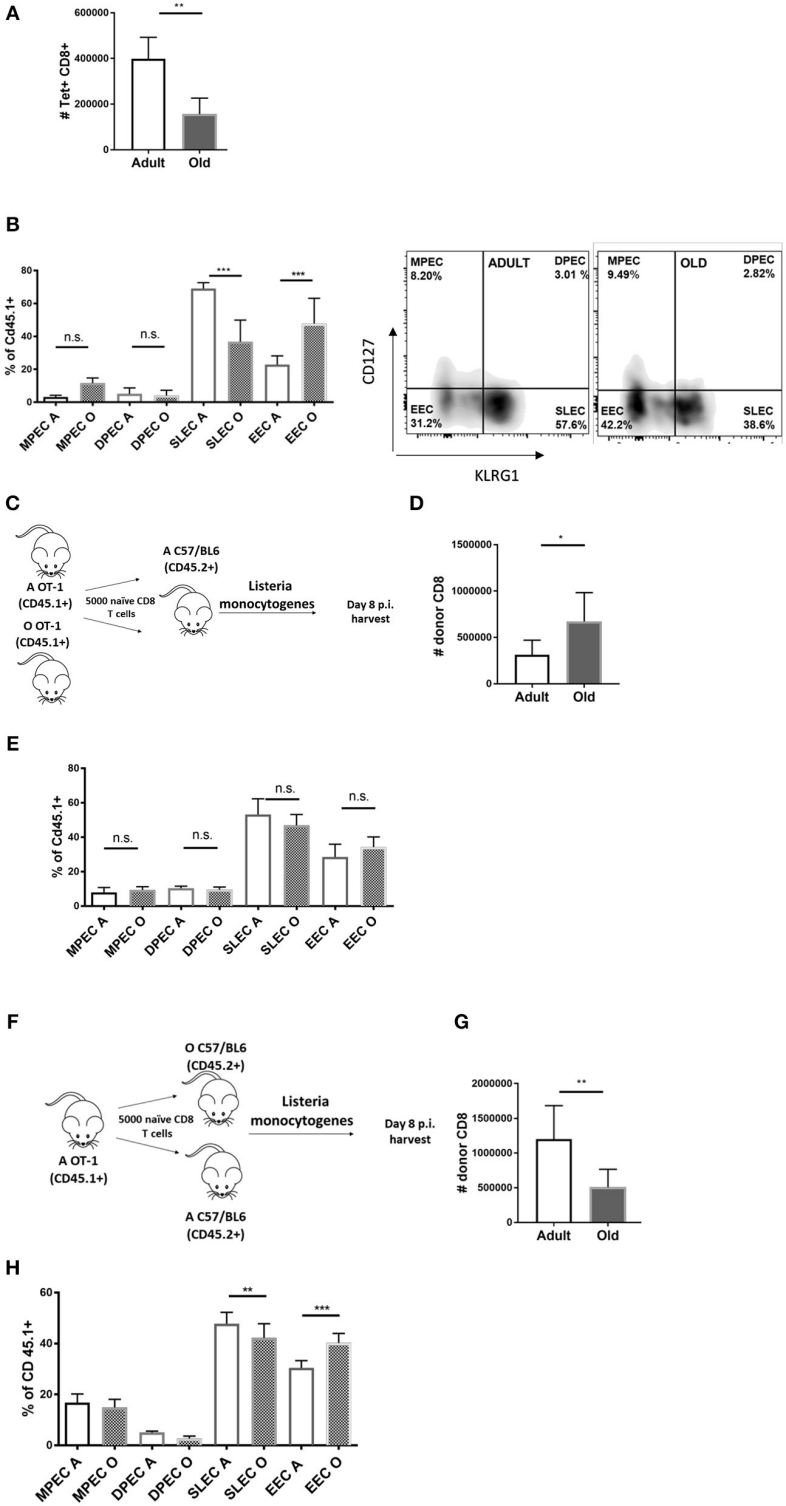
Impaired differentiation and expansion of CD8 T cells responding to *Listeria monocytogenes* infection in old mice is environmentally driven. Adult and old mice (*N* = 8) were inoculated intravenously (i.v.) with 10^4^ CFU Lm-OVA. On day 8 post Lm-OVA infection, at the peak of expansion of the T cell response for Lm-OVA mice were sacrificed, their spleens were homogenized and antigen specific response was measured by tetramer staining, old mice exhibited **(A)** decreased absolute numbers of Tet^+^ CD8 T cells in the spleen; and **(B)** reduced differentiation into SLEC (CD127-KLRG1+) and increased percentage of double negative EEC cells. **(C)** Naïve adult OT-1 CD8 T cells (CD45.1+) sorted from adult and old mice were transferred (*N* = 5,000) into adult C57/B6 (CD45.2+) mice and infected with Lm-OVA 24 h later. On day 8 post infection splenic T cells were analyzed by flow cytometry; **(D)** Old OT-I CD8 T cells expanded more than their adult counterparts when primed in the adult environment. **(E)** Terminal differentiation was equal between adult and old cells. **(F)** We performed the reciprocal transfer of adult OT-1 T cells into adult and old mice. **(G)** Expansion of adult OT-I T cells was decreased in old mice. **(H)** Terminal differentiation of adult OT-I T cells into SLECs was also decreased in the old environment (*p < 0.05, **p < 0.01, ***p < 0.001, ****p < 0.0001).

To study differentiation of old Ag-specific CD8 T cells, we used Lm expressing chicken ovalbumin protein (OVA) [Lm-OVA in the text (18)], allowing us to track Ag-specific CD8 T cells using the SIINFEKL:H-2K^b^ tetramer (Kb-OVA Tet). In our (11) and other hands (19) the Ag-specific CD8 response to *Listeria* peaks at days 7–8 p.i. and we have chosen these time points to analyse expansion and terminal differentiation of OVA-specific CD8 T cells. On day 8 post Lm-OVA infection, old mice exhibited decreased absolute numbers of Tet^+^ CD8 T cells in the spleen (Figure 1A) compared to adult mice, consistent with our prior data (11). Further, Ag-specific CD8 T cells in old mice showed reduced terminal differentiation into short-lived effector cells (SLECs) (Figure 1B). These SLECs can be identified by low expression of IL-7 receptor alpha chain (IL-7Rα; CD127) and high levels of KLRG1 (gating strategy in Figure S1A), an activating natural killer (NK) cell receptor (20). SLECs are more likely to terminally differentiate into effector CD8 T cells and ultimately die, unlike memory precursor effector cells (MPEC) that express CD127, but not KLRG1 (CD127+ KLRG1–). Recently, a new population of early effector cells (EECs) which lack expression of both CD127 and KLRG1 (CD127–KLRG1–) has been described (21), along with a small population of double positive effector cells (DPECs). Percentage of EECs (CD127–KLRG1–) was increased in old mice (Figure 1B) and these cells are known to exhibit plasticity and ability to give rise to both MPECs and SLECs (21). There was no difference in proportions of MPEC (CD127+KLRG1–) and DPEC (CD127+KLRG1+) cells between old and adult mice (Figure 1B). Analysis of absolute numbers of effector CD8 T cell subsets revealed that the SLEC subpopulation was vastly decreased in old mice (Figure S2). Previously, we have shown that CD8α DC defects in old mice contribute to poor CD8 T cell priming (17). To address whether T cell-intrinsic defects contributed to the age-dependent differences in expansion and differentiation of CD8 T cells we have enriched phenotypically naïve (CD62L^hi^CD44^lo^; >95% purity) CD8 T cells from spleens of adult (12 week) and old (18 month) mice and transferred them adoptively into adult (CD45.2) recipients. One day later, we infected the mice with Lm-OVA (schematic in Figure 1C). The expansion of the transferred old OT-I cells was slightly higher than their adult counterparts (Figure 1D) and their terminal differentiation was equal (Figure 1E), suggesting that any cell-intrinsic defects in old naïve CD8 T cells were below detection limits of our assay. The reciprocal transfer (schematic in Figure 1F) showed that as we previously published (17) expansion of OT-I T cells was decreased when primed in the old environment (Figure 1G). Terminal differentiation into SLECs was also decreased in the old environment (Figure 1H) although to a lower extent than in wild type old mice (Figure 1B). This partial replication of the SLEC defect observed in wt old mice in transferred OT-1 cells could be a consequence of fixing the T cell repertoire to single, high avidity TCR transgenic clonotype, a matter requiring further investigation. To investigate the kinetics of the age related environmental defect we have repeated this analysis on days 5, 7, and 9 p.i. SLEC differentiation of transferred adult OT-1 cells was lower on days 5 and 7 in old mice but tended to equilibrate by day 9 (Figure S3). However, the expansion defect in the old environment was still present on day 9 (Figure S4) nearing the contraction phase. Thus, it can be concluded that CD8 T cell expansion and differentiation are delayed and decreased in the old environment.

Overall, we found no difference in SLEC differentiation of old and adult OT-1 cells in adult mice (Figure 1E); this was also accompanied by increased expansion of old cells in the adult relative to old environment (Figure 1D). This indicates that old T cells maintain their expansion and differentiation potential when given proper molecular signals in the adult environment. We conclude that there is no observable intrinsic defect in CD8 T cells from old OT-1 mice while there is a clear defect in expansion and to a lesser extent SLEC differentiation, of adult OT-1 cells primed in aged mice.

### Antigen Specific CD8 T Cells Primed in the Old Environment Show Decreased T-bet Expression and Glucose Uptake

**Figure 2:**
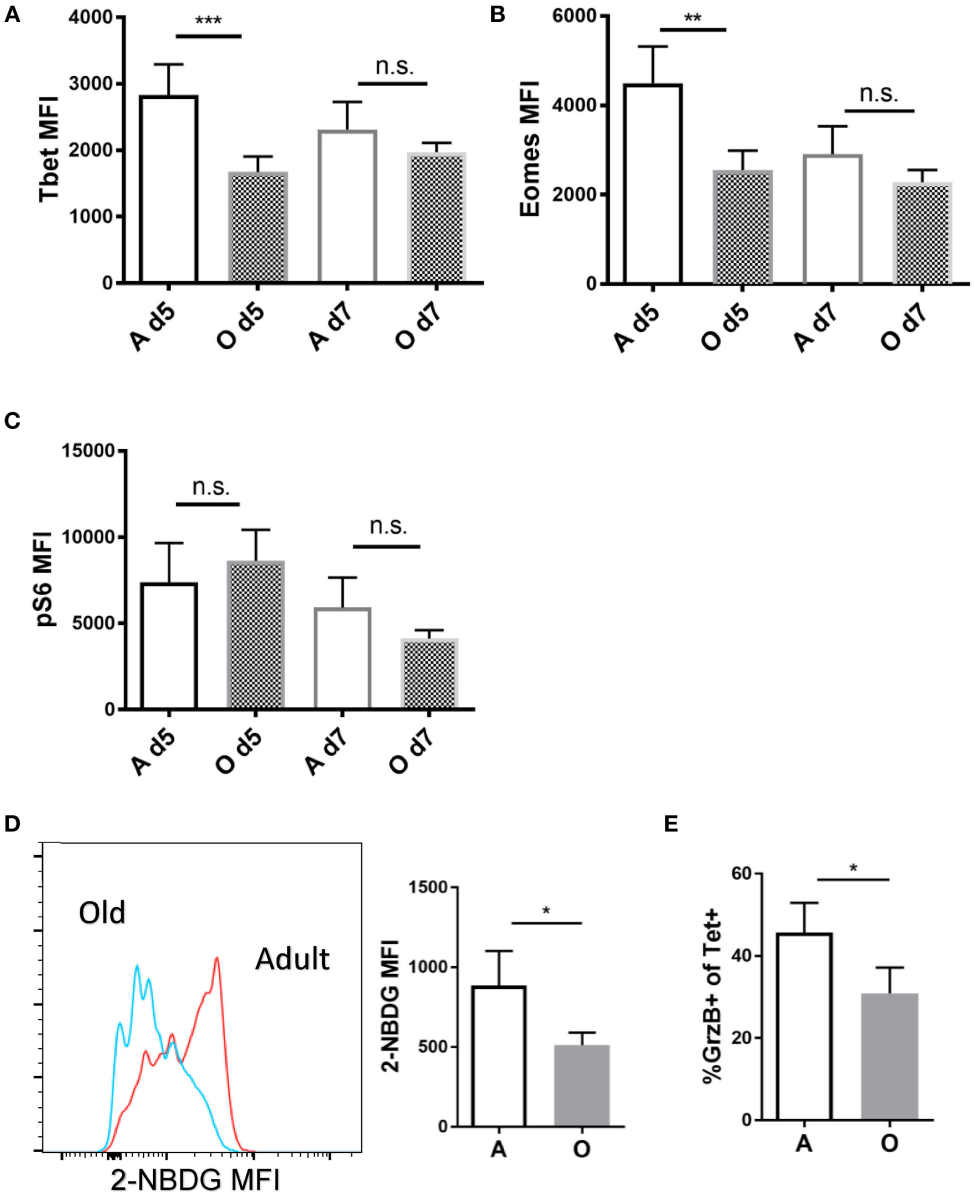
Antigen specific CD8 T cells primed in the old environment show decreased Tbet expression and glucose uptake. Sorted naïve adult OT-I CD8 T cells (CD45.1+, *N* = 5,000) were transferred into adult and old C57/BL6 (CD45.2+) mice (*N* = 10). Next day mice were inoculated intravenously (i.v.) with 10^4^ CFU Lm-OVA. On days 5 and 7 p.i. mice were sacrificed (*N* = 5), and expression of intracellular proteins Eomes, T-bet and pS6k was measured in OVA specific CD45.1+ CD8 T cells from the spleen by intracellular flow cytometric staining. **(A)** T-bet expression was significantly decreased in OT-1 CD8 T cells (CD45.1+) primed in the old environment on day 5. **(B)** Eomes expression was significantly decreased in OT-1 CD8 T cells (CD45.1+) primed in the old environment on day 5. **(C)** We found no difference in expression of ribosomal S6 kinase (pS6k). **(D)** Glucose uptake of transferred OT-1 cells was measured *in vivo* on day 5 using 2-NBDG, fluorescent glucose analog. 2-NBDG uptake was reduced on OT-1 CD8 T cells (CD45.1+) primed in the old environment. **(E)** Percentage of OT-1 T cells expressing effector molecule GrzB was reduced in old animals on day 5 (*p < 0.05, **p < 0.01, ***p < 0.001, ****p < 0.0001).

CD8 T cell effector differentiation is critically regulated by several transcription factors. T-bet, a master transcription factor encoded by the *Tbx21* gene, is necessary for the generation of functional effector CD8 T cells (22). T-bet expression leads to transactivation of genes encoding IFN-γ, granzyme B (GzB), and other potential targets (23). Eomes, another related T-box transcription factor, has been shown to be important for memory CD8 T cell development (24). Other transcription factors playing roles in effector CD8 differentiation include IRF4 (25) and BLIMP-1 (26, 27). We infected adult and old mice with Lm-OVA and measured transcription factor expression within Ag-specific effector CD8 T cells primed in adult or old environment by flow cytometry on days 5 and 7 of infection (representative FACS plots in Figure S1B). Day 5 p.i. was the earliest time point we could identify sufficient numbers of donor OT-1 cells (CD45.1+) to reliably measure expression of transcription factors and we have included one more time point at the peak of the response (day 7). Expression of Tbet was reduced on cells primed in the old environment on day 5 (Figure 2A) which was expected since SLEC differentiation was impaired in the old environment. Surprisingly expression of Eomes was also lower in old mice (Figure 2B) although previously we detected no differences in MPECs (Figure 1H). We found no differences in levels of IRF4 and BLIMP-1 between cells primed in the old and adult environment (Figure S5). Multiple signals converge to upregulate T-bet expression in activated CD8 T cells, and the mTORC1 pathway is critical in this process in both CD8 (28) and Th1 CD4 cells (29). T cell activation leads to the upregulation of glucose transport and a marked increase in aerobic glycolysis, thereby providing intermediate substrates for proliferation and production of effector molecules (30). This metabolic reprogramming is regulated by multiple signaling pathways including mTORC1, T-bet, and IRF4 (30). Of note, inhibition of the mTORC1 nutrient sensing pathway by rapamycin has been shown to decrease T-bet expression *in vitro* (28), demonstrating that the two pathways are connected. We explored whether phosphorylation of the mTORC1 regulated ribosomal S6 kinase (pS6k), glucose transporter Glut1 and corresponding uptake of glucose are maintained in CD8 T cells primed in the old environment, and whether any putative age-related differences in such expression may help explain impaired effector CD8 T cell differentiation with aging. We found no differences in pS6k (Figure 2C) or Glut1 levels (Figure S5), yet there was a pronounced decrease in the uptake of the glucose analog 2-NBDG (Figure 2D) in OT-1 cells primed in the old environment. 2-NBDG is a fluorescent analog of glucose used to quantify glucose uptake in activated CD8 T cells which is known to be higher in terminal effectors (31). Simultaneously, we found a decreased cytolytic potential in old Tet^+^ CD8 T cells, measured by the frequency of Tet^+^ cells that express GzB (Figure 2E), which we have previously shown to directly correlate to a decreased ability of old CD8 T cells to kill target cells (9). These results suggest that the priming environment in old mice fails to provide adequate molecular signals for the upregulation of key transcription and metabolic pathways needed for effector function and differentiation of CD8 T cells.

### Local Inflammation in Aged Secondary Lymphoid Tissue Is Significantly Reduced and Delayed

**Figure 3:**
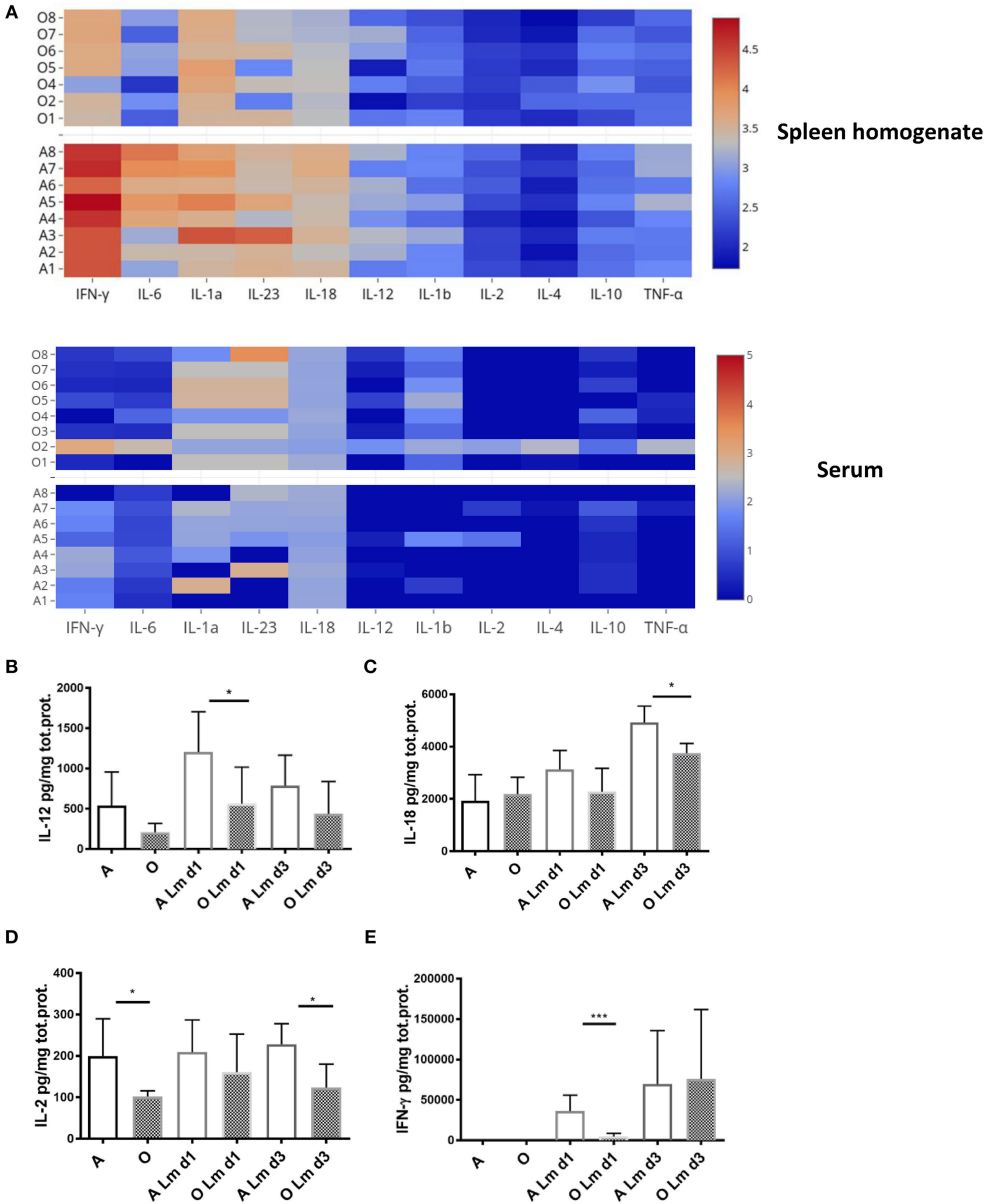
Old mice exhibit lower levels of multiple inflammatory cytokines early after infection with *Listeria monocytogenes*. Expression of multiple inflammatory cytokines was measured in spleen homogenates and serum of *Listeria* infected (10^4^ CFU i.v.) adult and old C57/BL6 mice (*N* = 8) on days 1 and 3 p.i. by Legendplex immunoassay. Spleens were homogenized in phosphate buffered saline containing protease inhibitor cocktail and 0.5% NP-40 detergent. **(A)** Heat map shows that the expression of all cytokines per mg total protein on day 1 p.i. was higher in spleen homogenate than serum while there was a decrease in multiple cytokines in spleen homogenates of old mice. **(B)** IL-12 was significantly decreased in old spleen homogenate on day 1 p.i. **(C)** IL-18 was significantly decreased in old spleen homogenate on day 3 p.i. **(D)** IL-2 was significantly decreased in old spleen homogenate of naïve mice and on day 3 p.i. **(E)** IFN-γ was drastically reduced in old mice on day 1 p.i. (*p < 0.05, **p < 0.01, ***p < 0.001, ****p < 0.0001).

*In vivo*, pro-inflammatory cytokines play a key role in regulating CD8 T cell activation. While brief exposure to peptide/MHC-I (signal 1) and co-stimulation (signal 2) is enough to induce several rounds of division in naïve CD8 T cells (32), pro-inflammatory cytokines (signal 3) are needed for clonal expansion and polarization toward specific fate and effector function as well as the expression of T-bet which is considerably enhanced and sustained in the presence of IL-12 (33). Other cytokines which can provide this third signal to naive CD8 T cells include type I interferons, IL-1, IL-18, and IL-33 [reviewed in Cox et al. (34)]. We measured various cytokines in the spleen homogenate and serum of old and adult mice during early priming against Lm (day 1 and 3) since it was shown that early inflammation directs CD8 T cell expansion and terminal differentiation (35). We found (Figure 3A) that levels of all measured cytokines, normalized per mg of total protein, were higher in the spleen homogenate compared to the serum. Spleen homogenates from old mice showed decreased overall inflammation on day 1 p.i. (Figure 3A) especially in the area of the heatmap including IFN-γ, IL-6, IL-1a, IL-23, IL-12, and IL-18; this decrease was not evident in serum.

Analysis of individual cytokine levels showed that IL-12 was induced robustly on day 1 in the adult mice but the induction was significantly blunted in the old mice (Figure 3B) and was still trending to be lower in the old on day 3. Similarly, levels of IL-18 were significantly higher in the adult compared to old spleens by day 3 (Figure 3C). IL-2 was lower in the old spleen homogenates at baseline (Figure 3D) and levels were not increased by Lm infection. By contrast, IFN-γ was significantly induced in the adult from several 100–~4,000 pg/mg (Figure 3E). This increase was curtailed and delayed in the old (Figure 3E) and IFN-γ levels were nearly 10-fold lower than adult on day 1. In contrast to the prevailing theory of inflammaging which refers to the chronic, low-grade inflammation that characterizes aging (36), these results imply that higher basal levels of inflammatory cytokines in serum (37) do not translate into adequate inflammatory response in the aged secondary lymphoid tissue and is insufficient to initiate completely developed adaptive immune responses. We have previously shown that there is no difference in bacterial burdens in spleens of old and adult mice (11), thus the decreased inflammatory reaction in spleens of old mice cannot be explained by less infection.

### Old naïve CD8 T Cells Proliferate and Upregulate Transcription Factors in Response to Inflammatory Cytokines *in vitro* Equally as Adult T Cells

**Figure 4:**
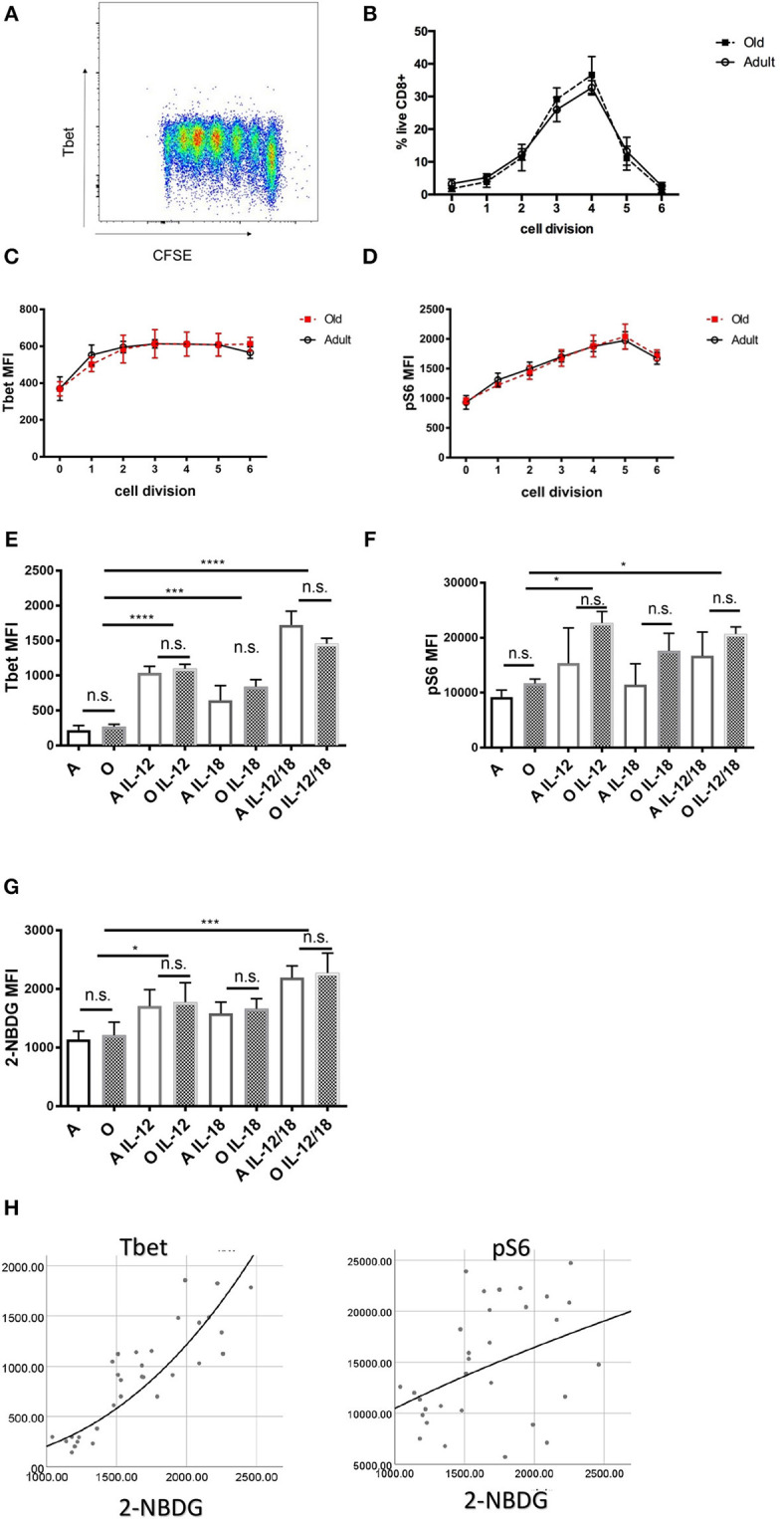
*In vitro* stimulated naïve CD8 T cells from old and adult wild type mice proliferate and express key transcription factor equally. Naïve (CD62L^hi^, CD44^lo^; >95% purity) CD8 T cells (20,000 cell/well) from adult and old C57/BL6 mice (*N* = 4) were activated *in vitro* with α-CD3 and α-CD28 beads in the presence of 10 U/ml rIL-2 and a bead to cell ratio of 1:1. **(A)** Representative flow cytometric dot plot of T-bet expression by proliferation peaks. **(B)** Adult and old naïve CD8 T cells proliferated equally in culture and expressed same levels of T-bet **(C)** and pS6k **(D)** in each of the subsequent cell division CFSE peaks. **(E)** T-bet was upregulated by addition of 10 ng/mL rIL-12 equally in adult and old CD8 T cells, addition of 10 ng/mL rIL-18 had a potentiating effect on Tbet upregulation. **(F)** pS6 was upregulated by IL-12 equally in adult and old CD8 T cells but with lesser synergistic effect with IL-18. **(G)** IL-12 individually and synergistically with IL-18 increased uptake of glucose analog 2-NBDG. **(H)** Multiple regression analysis with 2-NBDG (glucose uptake) as the dependent variable showed the strongest association with T-bet expression followed by pS6K and Glut1 (*p < 0.05, **p < 0.01, ***p < 0.001, ****p < 0.0001).

To rule out the possibility of TCR transgene artifacts, we tested whether old CD8 T cells from wildtype B6 mice also exhibit intrinsic defects. We used an *in vitro* culture system whereby naïve (CD62L^hi^, CD44^lo^; >95% purity) CD8 T cells from adult and old B6 mice were cultured with α-CD3 (TCR stimulation), α-CD28 (co-stimulation), and interleukin-2 (IL-2; for cell survival and proliferation) *in vitro*. Proliferation of stimulated T cells was measured by dilution of cytoplasmic dye CFSE (Figure 4A). We found that old CD8 T cells proliferated to same extent as adult (Figure 4B) and expressed equivalent amounts of T-bet 72 h after stimulation (Figure 4C). Phosphorylation of the S6 kinase was also equal in old and adult CD8 T cells (Figure 4D). Next, we examined how the addition of cytokines IL-12, IL-18, IFN-γ, and IL-2, which are each underproduced in the old spleen, affected T cell differentiation. IL-2 was previously shown to be essential for survival of activated T cells *in vitro* (38) but neither T-bet nor pS6K could be upregulated by increasing the amount of IL-2 from 10 to 100 U/ml. Addition of 20 ng/ml IFN-γ to the wells also had no effect on expression of T-bet or activation of S6K (Figure S6). By contrast, T-bet was upregulated by IL-12, and this activity was further enhanced with the inclusion of IL-18 (Figure 4E). This additive effect of IL-12 and IL-18 was equally pronounced in adult and old cells (Figure 4E). Similarly, there were no differences between adult and old cells in pS6K activation (Figure 4F), which was upregulated by IL-12 and IL-18 but without a synergistic effect. IL-12 and IL-18 also synergistically increased the uptake of the glucose analog 2-NBDG (Figure 4G), suggesting that these cytokines increase both the glucose flux and glycolytic rate, which are critical for effector function and proliferation. All these results highlight the critical finding that when provided with TCR agonist signal in the presence of adequate signal 3 cytokines, old CD8 T cells show no disfunction. To assess the relationship between glucose metabolism (2-NBDG uptake) and various transcription or metabolic pathway activation we have performed multiple regression analysis with 2-NBDG as the dependent variable. Expression of T-bet showed the strongest association with 2-NBDG uptake (Figure 4H) followed by pS6K and Glut1. No correlations were found with Eomes, IRF-4, and BLIMP-1 (Table S1). These results highlight the importance of T-bet in the programing of effector differentiation and metabolic switching of CD8 T cells and suggest that this pathway is not diminished in old naïve CD8 T cells.

### IL-12R KO OT-1 Cells Are Equally Dysfunctional in the Old and Adult Priming Environment

**Figure 5:**
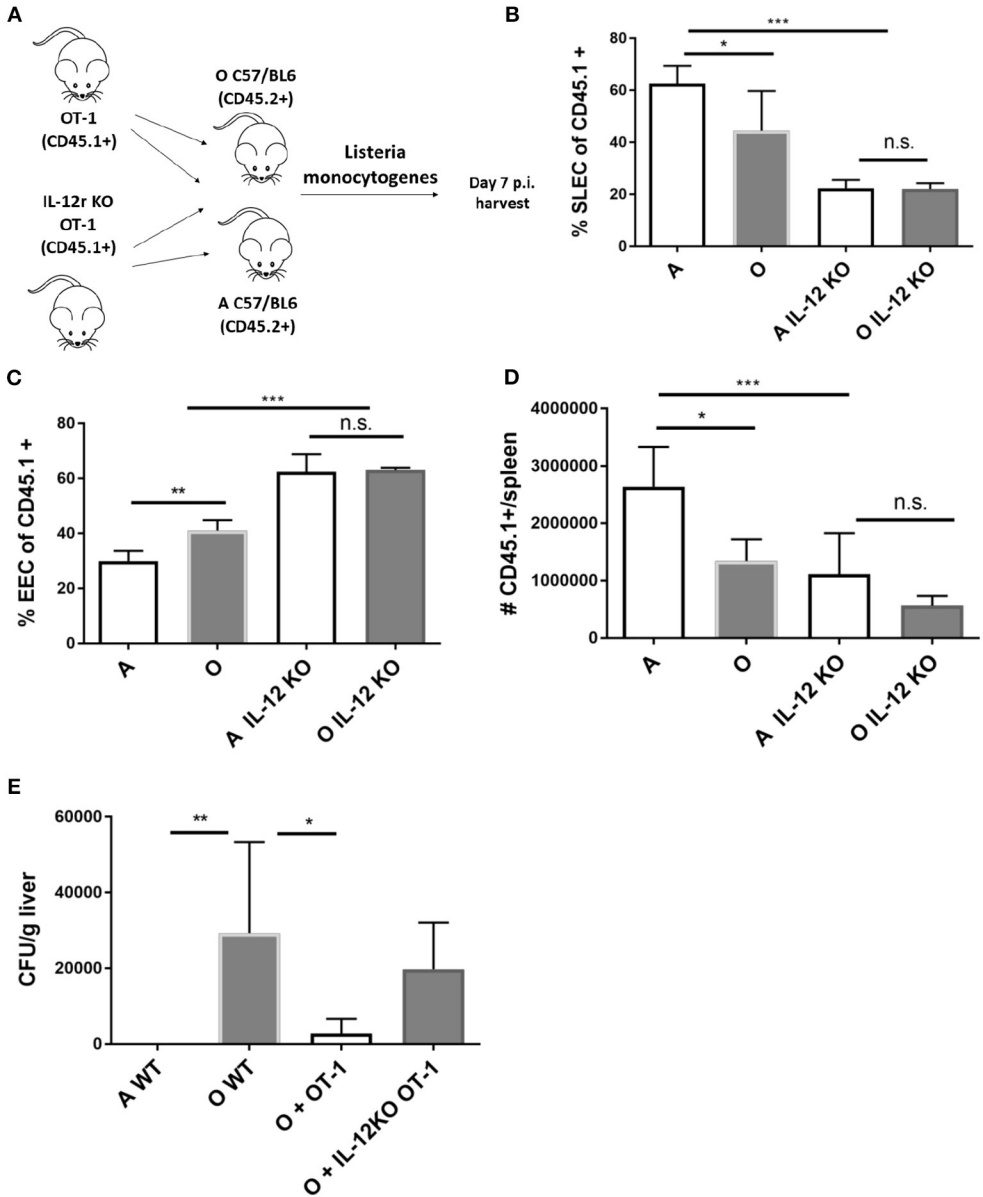
IL-12 KO OT-1 cells are equally dysfunctional in the old and adult priming environment. **(A)** Five thousand naïve IL-12R KO OT-1 CD8 T cells (CD45.1+) were transferred into adult and old mice C57/B6 (CD45.2+) mice (*N* = 5). Next day mice were inoculated intravenously (i.v.) with 10^4^ CFU Lm-OVA. On day 8 p.i. mice were sacrificed (*N* = 5), splenic T cells were analyzed by flow cytometry. **(B)** IL-12R KO OT-1 cells showed equally poor differentiation into SLECs in adult and old mice and were **(C)** predominantly of EEC phenotype. **(D)** Expansion of Ag specific cells was decreased in animals which received IL-12R KO cells. **(E)** Transfer of OT-1 transgenic CD8 T cells into old mice resulted in decreased bacterial burden in the liver but not if the OT-1 cells were IL-12R KO (*p < 0.05, **p < 0.01, ***p < 0.001, ****p < 0.0001).

To further investigate the *in vivo* role of IL-12 in age associated impairment of CD8 differentiation we generated OT-1 TCR transgenic mice deficient for IL-12 receptor by breeding them to IL-12Rb2-deficient (IL-12R KO) transgenic mice. We examined the response of Ag-specific CD8 T cells incapable of receiving IL-12 signals in the old and adult environment (Scheme in Figure 5A). IL-12R KO OT-1 cells in mice infected with Lm-OVA showed extremely low differentiation into SLECs (Figure 5B) confirming the role of IL-12 in effector differentiation as previously published (33). However, this impairment was equal in the adult and old animals (Figure 5B), and in both environments OVA-specific T cells favored the EEC fate (Figure 5C). Expansion of Ag specific cells was also decreased in animals which received IL-12R KO cells, again to the same extent in adult and old mice (Figure 5D). The lack of difference in expansion and differentiation of IL-12R KO OT-1 cells primed in adult and old environment points to a possible role of decreased IL-12 signaling in impaired effector CD8 T cell responses in the old environment. To further investigate the functional consequences of decreased IL-12 signaling we have measured liver burdens of *Listeria* in adult and old mice vs. old mice which have received transfer of 5,000 OT-1 cells or 5,000 IL-12R KO OT-1 cells. As we have previously published, old mice showed increased bacterial burdens in the liver which were reduced to the level of adult by addition of transgenic OT-1 T cells (Figure 5E). However, there was no significant decrease in bacterial burden if the old mice received IL-12R KO cells. These results showed the importance of the magnitude of the antigen specific CD8 T cell response as well as proper differentiation into terminal effector cells for adequate clearance of the bacteria from infected organs in old mice.

### *In vivo* Treatment With Recombinant IL-12 and IL-18 Repairs the Priming Defect in the Old Mice

**Figure 6:**
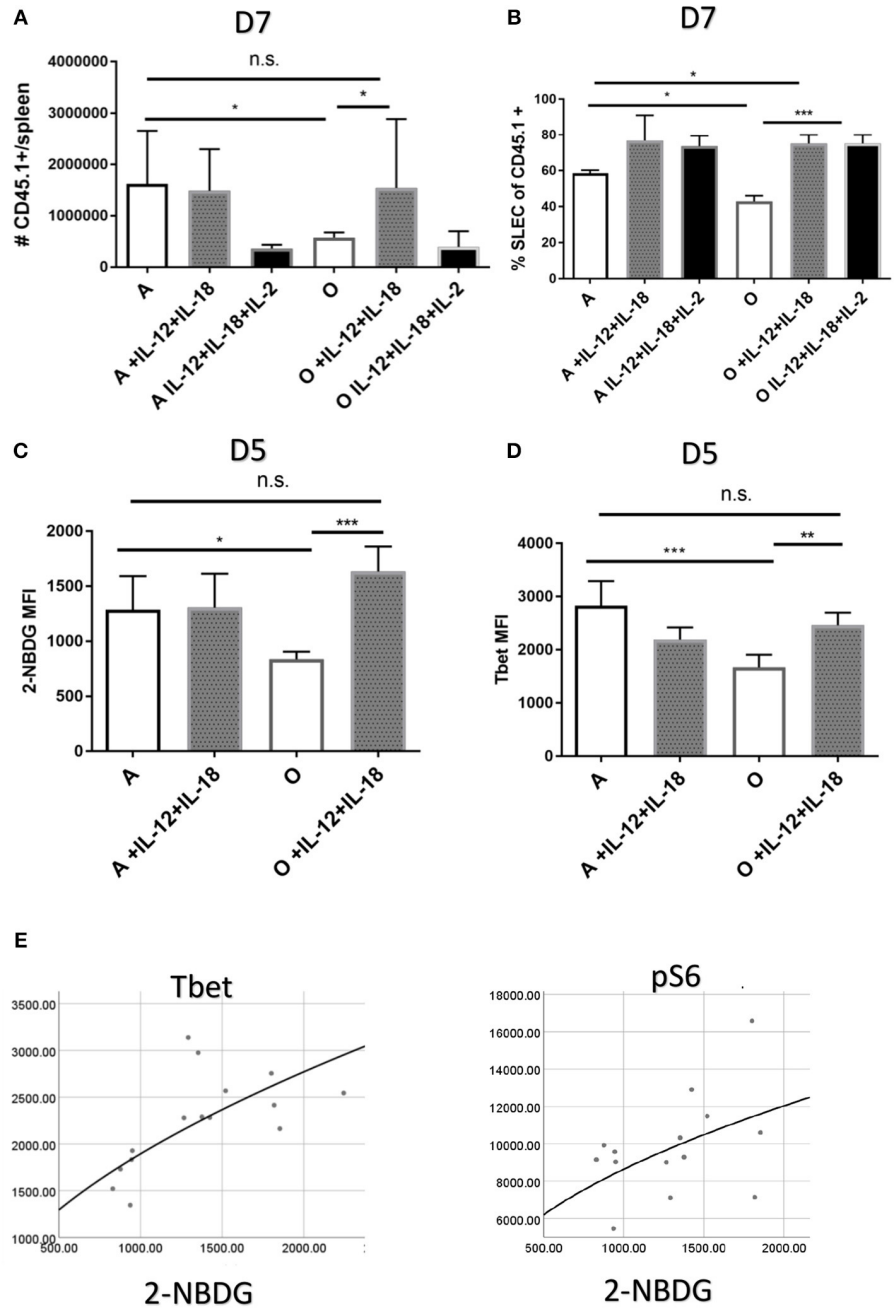
Treatment with recombinant IL-12 and IL-18 repairs the priming defect in the old organism. Adult and old C57/B6 (CD45.2+) mice (*N* = 10) received 5,000 adult naïve OT-1 CD8 T cells (CD45.1+). Next day mice were inoculated intravenously (i.v.) with 10^4^ CFU Lm-OVA and were consequently treated on days 4–6 p.i. with 0.5 μg of rIL-12 and rIL-18 with or without IL-2 cytokine antibody complexes. **(A)** IL-12 and IL-18 treatment increased the antigen specific response on day 7 in old mice but not in the adult while addition of IL-2 complex was inhibitory **(B)**. SLEC differentiation was significantly upregulated by cytokine treatment in both adult and old mice. **(C)** Glucose uptake early in infection (day 5) was upregulated by IL-12 and IL-18 only in old animals as well as **(D)** T-bet expression. **(E)** Multiple regression analysis with 2-NBDG (glucose uptake) as the dependent variable showed association only with T-bet and pS6k expression (*p < 0.05, **p < 0.01, ***p < 0.001, ****p < 0.0001).

Our *in vitro* results suggested that defective responses of aged CD8 T cells may be improved by *in vivo* induction or delivery of inflammatory cytokines. In fact, adjuvant properties of inflammatory cytokines have already been exploited to overcome some of the age related defects in CD4 T cell function of mice (39). Since our *in vitro* results showed the highest upregulation of Tbet through joint action of IL-12 and IL-18 we decided to test whether *in vivo* administration of these cytokines can improve or perhaps entirely restore the Ag specific response in old mice. Also, our initial *in vivo* results showed that while IL-12 supplementation alone (0.5 μg rIL-12/mice, days 4–6) did improve the SLEC differentiation in aged mice (Figure S7), it did not increase the expansion of Ag-specific cells (Figure S7). We included a group of mice which in addition to recombinant IL-12 and IL-18 received IL-2 cytokine-antibody complexes which were previously shown to enhance Ag specific responses (40). To avoid possible negative feedback mechanisms of inhibition of innate immunity by high doses of cytokines (41) we have chosen days 4–6 as timepoint for cytokine treatment. First, we have measured the number of Ag specific OT-1 T cells in adult and old treated mice on day 7 p.i. Treatment with IL-12 and IL-18 increased the antigen specific response in old mice and reconstituted it to the level of untreated adult (Figure 6A). In the adult, on the other hand, further addition of inflammatory cytokines IL-12 and IL-18 had no impact on the size of the Ag specific response (Figure 6A). Addition of the IL-2 complex to the cytokine mix did not further elevate the Ag specific response but rather had an inhibitory effect in both adult and old mice. This is of interest because the same cytokine or adjuvants that induce it (39) have been shown by Swain and colleagues to restore immune responsiveness in old CD4 T cells. Terminal differentiation into SLECs was also significantly improved in both adult and old mice (Figure 6B) by IL-12 and IL-18 treatment, whereas the addition of IL-2 had no additional effect on SLEC differentiation. These results show that the action of IL-12 and IL-18 was able to restore the expansion and differentiation of Ag specific cells in old mice to the level of the adult. To gain mechanistic insight of this improvement we performed an early (day 5) harvest after 2 cytokine injections and measured glucose uptake, expression of Glut1 and transcription factors T-bet, pS6k, Eomes, IRF4, and BLIMP-1. Treatment with recombinant IL-12/18 increased glucose uptake (Figure 6C) as well as expression of T-bet (Figure 6D) in old mice only. Multiple regression analysis showed that 2-NBDG as the dependent variable was best predicted by levels of T-bet expression (Figure 6E). The only other significant association was with mTORC1 activation [as measured by expression of downstream molecule pS6K (Table S2)]. These results point to Tbet as the single most important contributor, of the ones measured, to the observed functional defects in the old environment.

## Discussion

Upon primary challenge with intracellular infections, older individuals exhibit exacerbated disease symptoms and decreased survival (42). In mice, we have shown this to be in part due to decreased absolute numbers of Ag-specific CD8 T cells responding to infection, as well as decreased CD8 T cell production of cytokines and cytotoxic killing (9, 11), consistent with results obtained by others with influenza virus (43). However, these studies did not perform adoptive transfers, and therefore were unable to separate cell-intrinsic defects from those attributable to the aged environment.

Here, we present compelling evidence that old naïve CD8 T cells maintained their differentiation and metabolic functionality when given proper molecular signals; by contrast, naïve to effector transition of CD8 T cells was dramatically impaired in the old secondary lymphoid tissue irrespective of the age of the T cell. We found that T-bet expression and glucose uptake were reduced in old CD8 T cells, and both of these processes stand upstream of decreased effector molecule production and terminal differentiation, as previously suggested (30).

Previous research showing functional defects of aged CD8 T cells was potentially confounded by different subset representation of naïve and memory T cells in aged mice (44) and humans (45). Responses of memory and naïve T cells to antigen challenge are kinetically and functionally distinct (46). Therefore, only studies of minimally manipulated purified T cell subsets can detect age related functional defects on a per cell basis. Our results indicate that no defects exist in aged naïve CD8 T cells. More importantly, we show that impaired expansion and differentiation of naïve CD8 T cells in old organism can be entirely rescued by providing two T1-polarizing inflammatory cytokines, namely IL-12 and IL-18, which are underproduced in the old spleen. Soluble inflammatory signals provided through cytokines (signal 3) have a major impact on the magnitude of the CD8+ T cell and effector differentiation (47). Here, we show for the first time decreased local inflammatory response in aged secondary lymphoid tissue as evidenced by decreased level of multiple cytokines, namely IL-12, IL-18, IFN-γ, and IL-2. Importantly, none of the age-related cytokine defects other than IFN-γ were detected in the serum. Previous studies of innate immunity and aging have produced similarly paradoxical results. Serum increases in levels of proinflammatory cytokines, such as IL-6 and TNF-a and acute-phase reactants have been termed inflammaging (37), yet there is convincing evidence that microbial product-induced cytokine production of innate cells, such as macrophages and dendritic cells, is decreased [reviewed in Montgomery and Shaw (48)]. Thus, our results suggest that tissue homogenates are more informative than serum in studying cytokine responses in aged animals. Furthermore, we have identified IL-12 and IL-18 as two key cytokines which upregulated T-bet, glucose uptake, terminal differentiation, and expansion of Ag specific CD8 T cells in old mice. Data were consistent with corroborating results from IL-12 receptor deficient CD8 T cells, that exhibited equal impairment in old and adult mice, further highlighting the central role of IL-12 in these processes. IL-12 was previously shown to prolong the division of activated CD8 T cells (40) and recombinant IL-12 (rIL-12) injection has potent adjuvant activity and anti-tumor CD8 T cell mediated immunity (49). IL-18 is known to synergize with IL-12 in inducing effector function of memory CD8 T cells (34) and in our hands these two cytokines had a strong effect on induction of T-bet in TCR activated naïve CD8 T cells both *in vivo* and *in vitro*. Upregulation of T-bet by IL-12 was previously shown to drive SLEC differentiation (33). Other transcription factors such as IRF4 (50), and Blimp-1 (26) share redundant roles with T-bet in promoting CD8 T cell effector function and terminal differentiation and could also be contributing to age related dysfunction of CD8 T cells. Regardless of functional overlap, our multiple regression analysis showed that unlike other measured transcription factors, T-bet expression levels were more predictive of increased glucose uptake [a prerequisite for effector function (51)].

In conclusion, we showed that differentiation and expansion defects of Ag specific CD8 T cells in the old mouse are driven by defects in the priming environment, namely decreased production of IL-12 and IL-18, which in the old spleens resulted in insufficient metabolic and transcriptional programming of CD8 T cell effector function. These results might be relevant to vaccination of elderly humans; impaired T cells response to vaccination in older adults was previously described (52) and could be improved with addition of an adjuvant system (ASO1) in a IL-12 and IL-18 dependent fashion (53). Experiments are in progress to assess translational potential of these findings.

## Experimental Procedures

### Mice

Adult (12 weeks old) and old (19–21 months old) C57BL/6 (B6) mice were obtained from the Jackson Laboratories (Bar Harbor, ME) and the NIA breeding colony (Charles River Laboratories), respectively. (B6.OT-I.Rag-KO x B6.Ly5-1) F1 mice were bred in-house from stocks of B6.OT-I.Rag-KO and B6.SJL mice, purchased from Taconic Farms (Tarrytown, NY). IL-12Rb2-deficient (IL-12R KO) mice were obtained from Jackson Laboratory. All experiments were performed with groups of 4–8 animals. Mice were maintained in specific pathogen free conditions at the University of Arizona, and experiments were conducted under guidelines and approval of the Institutional Animal Care and Use Committee at the University of Arizona.

### Adoptive Transfers

For adoptive transfer experiments into adult or old C57/BL6 recipients (CD45.2+), naïve OT-I CD8 T cells (CD45.1) were enriched from adult mice. Five thousand cells were transferred i.v. and hosts were infected with Lm-OVA the following day. For adoptive transfer of adult or old OT-I cells into adult recipients, naïve CD8 T cells were enriched as for cell culture from 3 or 18 mo. old OT-I mice. Five thousand cells were transferred into adult B6 recipients who were infected with Lm-OVA the following day. Transferred cells were later identified with congenic marker (CD45.1) and tetramer staining.

### Infections and Cytokine Treatments

*L. monocytogenes*-OVA (Lm-OVA), Lm strain 10403 engineered to express chicken ovalbumin was generously provided by Dr. Hao Shen (University of Pennsylvania, Philadelphia, PA). Infections were performed as previously described (11). Briefly, Lm-OVA bacteria were grown overnight in BHI, mice infected with 1–3 × 10^4^ colony forming units (CFU); 100 μl sterile PBS i.v. and the number of inoculated Lm validated *post hoc* by plating serial dilutions of the injected solution on BHI agar. Infected mice were treated on days 4–6 p.i. with 0.5 μg of rIL-12 and rIL-18 (Biolegend) with or without IL-2 cytokine antibody complexes (300 ng IL-2–10 μg S4B6 antibody) prepared as previously described (40).

### Flow Cytometry

Samples were prepared as previously described (11) and single-cell suspensions in 100 μl PBS-2% FBS (FACS wash) transferred to a 96-well round-bottom plates for intracellular cytokine staining or tetramer (Tet^+^) staining analysis. Kb-OVA tetramers conjugated to either APC or PE were generously provided by the NIH Tetramer Core Facility (Atlanta, GA) in support to the NIH/NIAID contract HHSN27220110017C. The following antibodies were used for flow cytometric staining: CD3 (17A2), CD8a (S3-6.7), CD45.1 (A200), CD127(A7R34), KLRG1 (2F1), T-bet (4B10), Eomes (Dan11mag), Blimp-1 (SE7), GrzB (QA16A02), pS6 (cupk43k), glut1 (EPR3915), IRF-4(IRF4.3E4). Samples were analyzed on a 4-laser custom Fortessa cytometer (BDIS, Sunnyvale, CA), using the DiVA acquisition (BDIS) and FlowJo (Treestar, Ashland, OR) analysis software.

### Cytokine Measurements

On days 1 and 3 after infection spleens were homogenized in phosphate buffered saline in the presence of protease inhibitor cocktail (Sigma) and 0.5% NP-40 detergent. Concentration of cytokines IL-2, IL-12, IL-10, IL-1a, IL-1b, IL-27, IL-33, and IL-23 were measured by Legendplex flow cytometry kit (Biolegend). Concentration of IL-18 was measured by ELISA (Thermo Fisher Scientific, Waltham, MA).

### Intracellular Staining

Samples were stimulated and prepared as previously described (11). Cells were stained with surface antibodies, and then fixed and permeabilized using either the FoxP3 Fix/Perm kit (eBioscience) or the Fix/Perm kit (BD Bioscience) followed by staining with intracellular antibodies. Protein expression was measured by calculating the mean fluorescence intensity (MFI).

### *In vivo* Labeling of Glucose Uptake Using 2-NBDG

On day 5 post Lm injection mice received retroorbital injection of 100 μL of 10 mM 2-NBDG solution in PBS, were sacrificed 15' later and spleens processed and stained with surface antibodies for flow cytometric analysis.

### Cell Culture

Splenic CD8 T cells were magnetically enriched using an AutoMACS pro (Miltenyi Biotec) to >95% purity per manufacturer's instructions. The cells were fluorescein-labeled with 5 μM carboxyfluorescein succinimidyl ester (CFSE) at 37° for 10', washed 3x with RPMI-1640 medium supplemented with 2-mercaptoethanol, L-glutamine, pyruvate, non-essential amino acids, and 10% fetal bovine serum (abbreviated as RP10 in the text). The cells were cultured at 2 × 10^5^ cells/ml at a 1:1 ratio with α-CD3/α-CD28 immobilized on beads (Miltenyi), in the presence of combinations of 10 U/ml rIL-2, 10 ng/ml rIL-12 (Biolegend), and 10 ng/ml rIL-18 (Biolegend), as indicated, for up to 3 days. On each day of analysis, cells were stained for viability (LIVE/DEAD reagent, Invitrogen), as well as with surface and/or intracellular antibodies.

### Statistical Analysis

All analyses were performed using GraphPad Prism software (GraphPad Software Inc, San Diego, CA) or SPSS (IBM Corp. Armonk, NY). Differences were calculated by Student's *t*-test, Mann Whitney *U*-test or one-way ANOVA, and Kruskal-Wallis test depending on data distribution. Probability values of *p* < 0.05 were considered significant. Multiple regression analysis was performed by SPSS. The following notations have been used throughout the figure legends to denote *p* values: ^*^p < 0.05, ^**^p < 0.01, ^***^p < 0.001, ^****^p < 0.0001. All data denote mean ± SEM unless otherwise noted. Symbols indicate individual mice. All data are pooled results from at least two independent experiments.

## Data Availability

All datasets generated for this study are included in the manuscript and/or the Supplementary Files.

## Ethics Statement

Mouse studies were carried out in strict accordance with the recommendations in the Guide for the Care and Use of Laboratory Animals of the National Institutes of Health. Institutional Animal Care and Use Committee at the University of Arizona (IACUC #08–102, PHS Assurance Number: A3248-01) approved all protocols used in this report. Euthanasia was performed by isoflurane overdose.

## Author Contributions

MJ, MS, and JN-Z designed experiments. MJ, HT, KR, and MS performed experiments. MJ analyzed the data. MJ and JN-Z wrote the manuscript. JN-Z administered the project.

### Conflict of Interest Statement

The authors declare that the research was conducted in the absence of any commercial or financial relationships that could be construed as a potential conflict of interest.

## Abbreviations

Ag: antigen
EE: early effector T cells
Lm: *Listeria monocytogenes*
*MPEC*: memory precursor effector cells
p.i.: post infection
SLEC: short-lived effector cells
TCR: T cell receptor.
