# Corrigendum: Defective Transcriptional Programming of Effector CD8 T Cells in Aged Mice Is Cell-Extrinsic and Can Be Corrected by Administration of IL-12 and IL-18

**DOI:** 10.3389/fimmu.2020.01367

**Published:** 2020-07-28

**Authors:** Mladen Jergović, Heather L. Thompson, Kristin R. Renkema, Megan J. Smithey, Janko Nikolich-Žugich

**Affiliations:** ^1^Department of Immunobiology and the University of Arizona Center on Aging, University of Arizona College of Medicine-Tucson, Tucson, AZ, United States; ^2^Biomedical Sciences Department, Grand Valley State University, Allendale, MI, United States

**Keywords:** aging, cytotoxic T cells, inflammation, cytokines, bacterial infection

In the original article, we neglected to include the funder National Institute of Health, R01 AG020719 and P01 052359 to Janko Nikolich-Žugich. The updated **Funding** statement appears below. The authors apologize for this error and state that this does not change the scientific conclusions of the article in any way. The original article has been updated.

## Funding

This work was supported by USPHS awards NIH/NIAID HHSN27220110017C, R01 AG020719, and P01 052359 to JN-Ž from the National Institute of Health. The funders had no role in study design, data collection and analysis, decision to publish, or preparation of the manuscript.

