# Supplementary material for: Defective Transcriptional Programming of Effector CD8 T Cells in Aged Mice Is Cell-Extrinsic and Can Be Corrected by Administration of IL-12 and IL-18
Source: Front Immunol. 2019 Sep 18;10:2206. doi: 10.3389/fimmu.2019.02206 (PMC6759569; doi:10.3389/fimmu.2019.02206)
Supplement: Supplementary file 1 [file Data_Sheet_1.PDF]

A

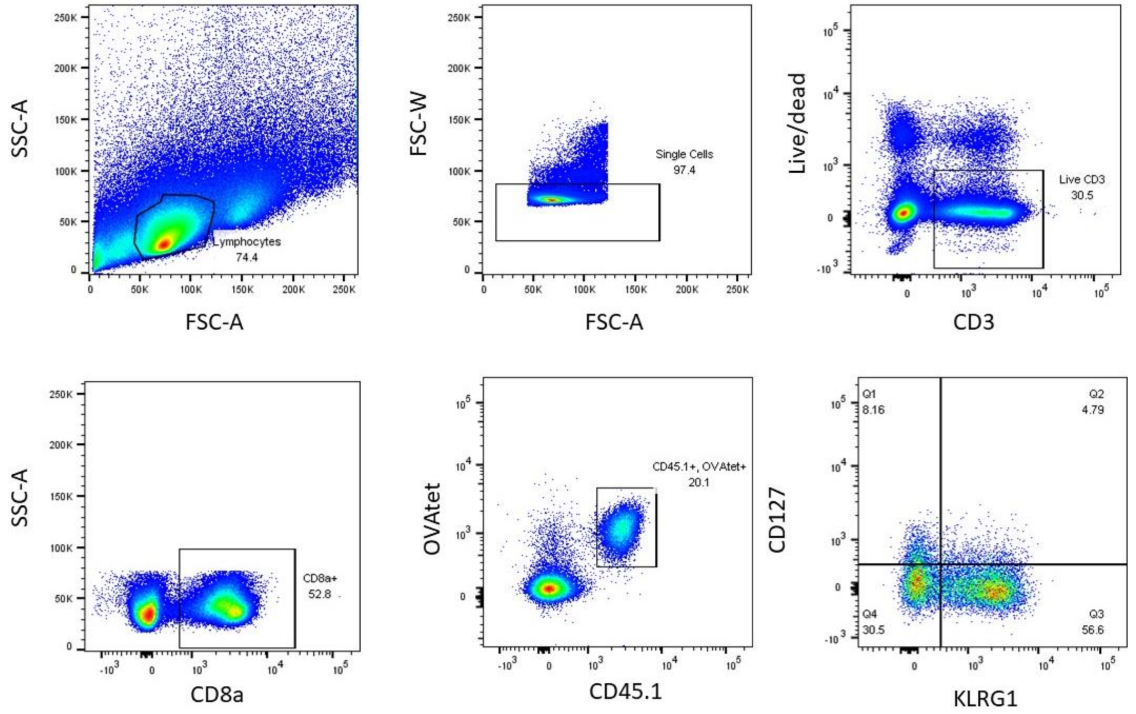

B

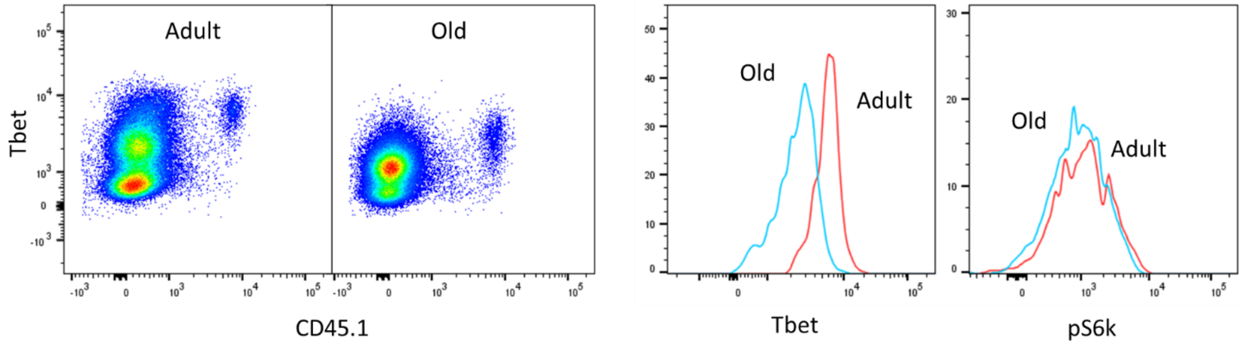

Figure S1. A) FACS gating scheme for identifying OT-1 cells and their differentiation into terminal effectors (CD27-KLRG1+) **B)** FACS plots of intracellular protein expression of T-bet and pS6k in old and adult mice on day 5 p.i.

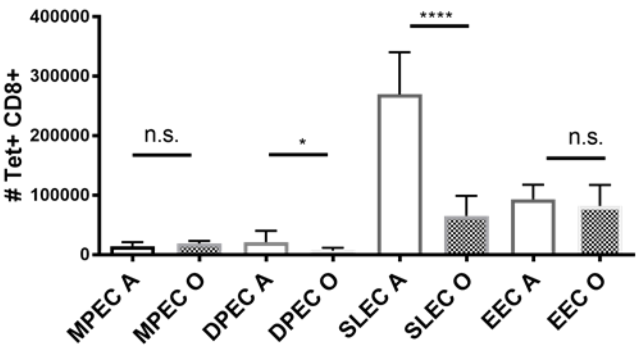

Figure S2. Absolute numbers of OVA specific effector CD8 T cells in adult and old mice on day 8 of Lm-OVA infection

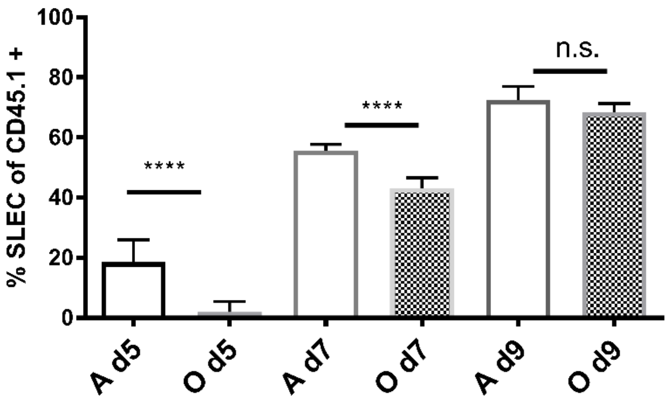

Figure S3. Kinetics of SLEC phenotype in the adult and old mice

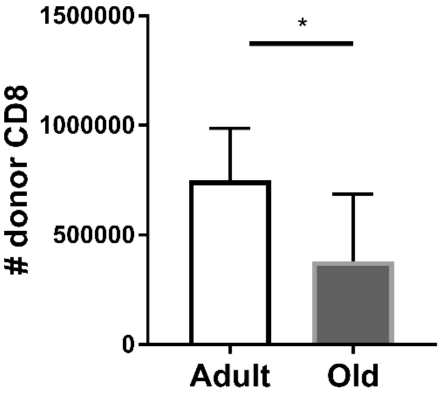

Figure S4. Absolute number of transferred OT-1 cells was lower in old than adult mice on d9 p.i.

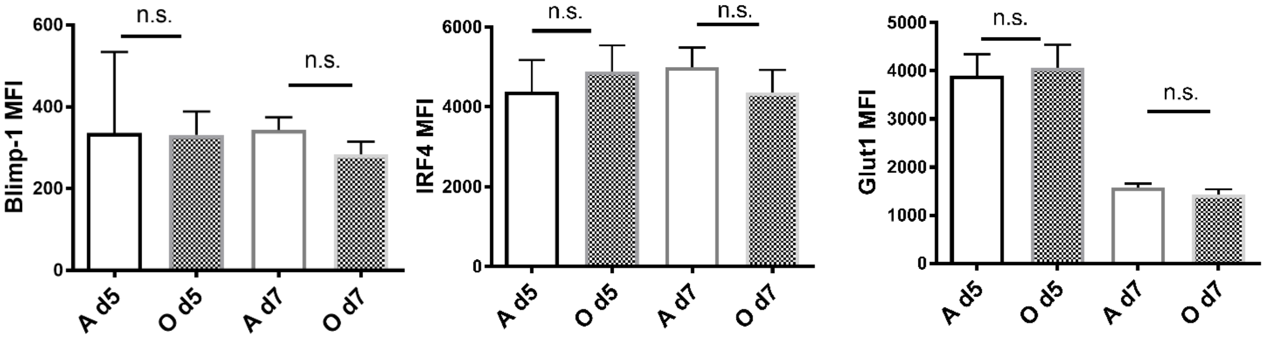

Figure S5. Expression of transcription factor BLIMP-1 and IRF-4, as wells as glucose transporter Glut1 was equal between cells primed in old and adult environment.

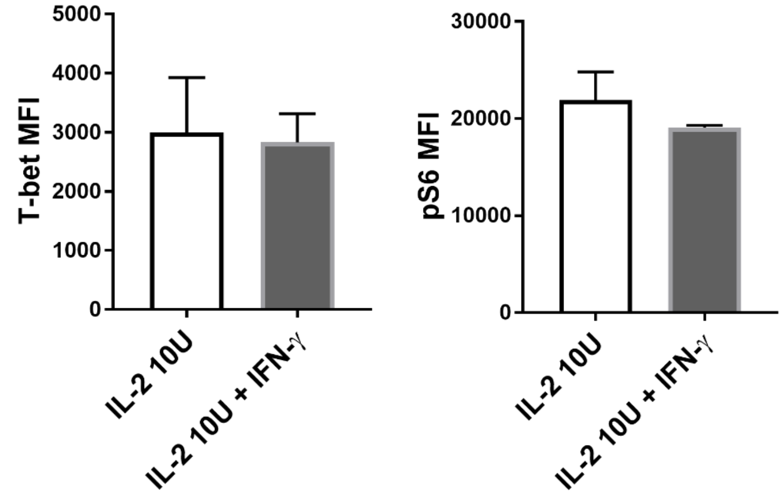

Figure S6. Expression of T-bet and pS6 was unaltered by addition of 20 ng/ml IFN-γ in both adult and old stimulated naïve CD8 T cells.

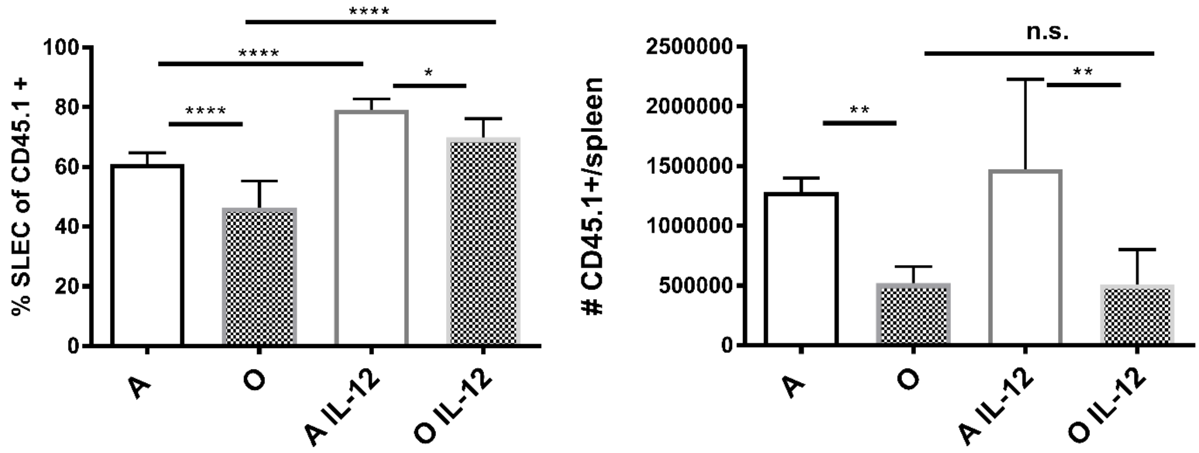

Figure S7 In vivo supplementation of IL-12 (0.5 µg/mice) alone corrected the SLEC differentiation defect in old mice but had no effect on the absolute numbers of transferred OT-1 cells (CD45.1+)

**2-NBDG**	**Unstandardized B**	**Std. Error**	**Std. Beta**	**t**	**Sig'**
**Tbet**	2.564	.261	.873	9.8414	**0.000**
**pS6**	.703	.273	.426	2.576	**0.015**
**Glut-1**	1.726	.202	.842	8.542	**0.000**
**Eomes**	-.003	.844	-.001	-.004	.997
**IRF-4**	-.092	.197	-.128	-.466	.649
**Blimp-1**	-.072	.306	-0.43	-.235	.816

Table S1 Multiple regression analysis of *in vitro* expression of transcription factors with 2-NBDG as the dependent variable showed highest association with levels of T-bet

**2-NBDG**	**Unstandardized B**	**Std. Error**	**Std. Beta**	**t**	**Sig'**
**Tbet**	.549	.153	.706	3.595	**0.003**
**pS6**	.477	.220	.515	2.166	**0.049**
**Glut-1**	.190	.225	.228	.845	.413
**Eomes**	.258	.281	.247	.981	.375
**IRF-4**	-.092	.197	-.128	-.466	.649
**Blimp-1**	-.265	.226	-.309	-1.171	263

Table S2 Multiple regression analysis of *in vivo* expression of transcription factors with 2-NBDG as the dependent variable showed highest association with levels of T-bet
